# Country- and app-level factors affecting the adoption and evaluation of COVID-19 mobile apps

**DOI:** 10.1186/s12889-022-14918-8

**Published:** 2022-12-31

**Authors:** Yi Wu, Qianying Ye, Fei Shen, Zhian Zhang, Crystal Li Jiang

**Affiliations:** 1 School of Communication and Design, Sun Yat-sen University, Guangzhou, China; 2 Department of Public and International Affairs, City University of Hong Kong, Hong Kong, China; 3 Department of Media and Communication, City University of Hong Kong, Hong Kong, China; 4 School of Journalism, Fudan University, 400 Guoding Rd., Yangpu District, 200433 Shanghai, China

**Keywords:** COVID-19 app, Adoption, Evaluation

## Abstract

**Background:**

Countries across the globe have released many COVID-19 mobile apps. However, there is a lack of systematic empirical investigation into the factors affecting the adoption and evaluation of COVID-related apps. This study explores what factors at the country level and the app levels would influence the adoption and evaluation of COVID-19 apps.

**Methods:**

We collected data on 267 COVID-19 apps in App Store and Google Play. The number of installs, ratings, reviews and rating scores were used as indicators of adoption and evaluation. Country-level predictors include the number of infected cases and the political system (i.e., democratic vs. non-democratic). App-level predictors include developer (i.e., government vs. non-government) and functions. Four app functions were coded for analysis: providing health information, contact tracing, home monitoring, and consultation. Negative binomial regression and OLS (Ordinary Least Square) regression were used to analyze the data.

**Results:**

Our analyses show that apps developed by countries with more infected cases (B = 0.079, CI (Confidence Interval) = 0.000, 0.158; *P* = .049) and by non-governmental institutions (B=-0.369, CI=-0.653, -0.083; *P* = .01) received more positive rating scores. Apps with home monitoring function received lower rating scores (B=-0.550, CI=-0.971, -0.129; *P* = .01). Regarding adoption, apps developed by governments were more likely to be installed (IRR (Incident Rate Ratio) = 8.156, CI = 3.389, 19.626; *P* < .001), to be rated (IRR = 26.036, CI = 7.331, 92.468; *P* < .001), and to receive user comments (IRR = 12.080, CI = 3.954, 37.568; *p* < .001). Apps with functions of contact tracing or consultation were more likely to be installed (IRR = 4.533, CI = 2.072, 9.918; *p* < .001; IRR = 4.885, CI = 1.970, 12.111; *p* < .001), to be rated (IRR = 11.634, CI = 3.486, 38.827; *p* < .001; IRR = 17.194, CI = 5.309, 55.680; *p* < .001), and to receive user comments (IRR = 5.688, CI = 2.052, 5.770; *p* < .001; IRR = 16.718, CI = 5.363, 52.113; *p* < .001). Apps with home monitoring functions were less likely to be rated (IRR = 0.206, CI = 0.047, 0.896; *P* = .04) but more likely to receive user comments (IRR = 3.874, CI = 1.044, 14.349; *P* = .04). Further analysis shows that apps developed in democratic countries (OR (Odd Ratio) = 3.650, CI = 1.238, 10.758; *P* = .02) or by governments (OR = 7.987, CI = 4.106, 15.534, *P* < .001) were more likely to include the function of contact tracing.

**Conclusion:**

This study systematically investigates factors affecting the adoption and evaluation of COVID-19 apps. Evidence shows that government-developed apps and the inclusion of contact tracing and consultation app functions strongly predict app adoption.

## Introduction

COVID-19 appeared in late 2019 in China and is still impacting the world. Since the virus has created an unprecedented health crisis worldwide, efforts were made to reduce the risk of COVID-19 by governments and other institutions. Mobile apps are considered practical and valuable tools for managing health [1, 2]. More than 5 billion people are currently using mobile phones [3], and 204 billion apps were downloaded in 2020 [4]. Apps are used worldwide as tools for various purposes in current days. By now, many COVID-19 mobile apps have been developed as an anti-epidemic measure and are available in app stores, including Google Play (Android) and Apple App Store (iOS).

### Objective

This study aims to analyze currently existing COVID-19 apps systematically. Specifically, we examine what country-level and app-level factors are associated with adopting and evaluating the apps. This study could provide a global perspective on COVID-19 app usage during the pandemic.

### Related works

As of now, studies on COVID-19 apps are surrounding three areas. A majority of works focus on contact tracing. Contact-tracing apps have emerged in response to the outbreak of COVID-19. Apps with contact-tracing functions can assist identification of people who may have contact with an infected person and collect further information about these contacts [5].

First, scholars are interested in the architectures of contact-tracing apps, namely centralized, decentralized, or hybrid design [6, 7]. A centralized architecture means that the government authority controls personal data collection, while the decentralized approach means that personal data is stored only on personal devices. Therefore, a trade-off exists between data privacy and effective disease control by the government [6]. The development and deployment of such architectures and technologies are under ongoing discussion.

Second, there are concerns about the ethical problems of contact-tracing apps [8, 9]. These questions are such as in the context of the COVID-19 pandemic, whether the previous data protection criteria can be justifiable; the trade-off between liberty and privacy; whether the installation of these apps should be compulsory; whether data should be deleted at the end of the epidemic; etc. [9]

Third, people’s attitude toward contact-tracing apps is another critical aspect. Using experiments or surveys, researchers assess attitudes toward contact-tracing apps, including experience of use, privacy concern, and willingness to adopt [6, 10–17]. Studies found that people hold different attitudes toward these apps. Some refuse to use them, while some voluntarily accept surveillance or even become surveillance agents to monitor the behavior of others to obtain a safe living environment [18].

Another group of studies is from the technical perspective. These articles review existing COVID-19 apps in terms of features and functionalities [1, 19–22]. For example, Ming et al. reviewed the COVID-19 apps launched in the early days of the pandemic and found that most of the apps incorporate the functions of case mapping or home monitoring surveillance [19]. Noronha et al. reviewed the COVID-19 apps in Canada and assessed the goals and characteristics of these apps [22].

Finally, there are many articles about the assessment of COVID-19 apps. Scholars use specific rating scales to assess the quality, functionality, design, engagement, and other features of COVID-19 apps [21, 23, 24]. These studies mainly aim to provide overviews of existing apps for the further development of new apps and suggest what should be included as vital features [21, 23].

### Research gap

For now, the function of contact tracing is the focus of research on COVID-19 apps. For instance, attitudinal research is mainly limited to contact-tracing apps. These studies use experiment [13] or survey [6, 11] to assess people’s attitudes, restricting the findings to the individual and the regional level. Besides contact tracing, there are many other functions COVID-19 apps possess. These apps, other than contact-tracing apps, have received less scholarly attention. However, learning what kinds of COVID-19 apps have received more adoptions and higher evaluations is essential. It might help scholars and health professionals enhance their understanding of app use in a crisis.

The current study will provide a global perspective on the adoption and evaluation of COVID-19 apps. Using comprehensive data from Google and Apple app stores, we can assess people’s adoption and evaluation based on the number of downloads, ratings, reviews, and rating scores of the apps. Our study will extend previous studies on COVID-19 apps to a much broader perspective as we have collected almost all recent existing COVID-19 apps worldwide, regardless of language and country. In addition, not only are the app-level factors, such as features and functions, decisive for the adoptions and evaluations, but also country-level factors would be critical potential predictors. Our data allow us to access such macro-level factors, rarely studied previously.

### Rationale and hypotheses

According to a European study, people’s perception of benefits, self-efficacy, and perceived barriers are all associated with willingness to use the COVID-19 contact-tracing apps [16]. Beyond individual factors, country-level factors might also be important predictors of the adoption and evaluation of COVID-19 apps. For example, regarding the political system, people in democratic countries concern more about individual privacy and state surveillance [25]. Therefore, democratic societies might have a lower adoption rate of apps that need to collect personal data than non-democratic societies. However, many democratic countries are developed countries that might have a higher level of digital technology. People might rely more on technologies to obtain information and trace cases and contacts [11]. Besides, the number of infected patients in a country might influence the adoption of COVID-19 apps. A large number of infected cases goes with a higher perception of risk and severity. Thereby, the adoption rate might increase. Based on the discussion above, we are concerned about the macro-level predictors of COVID-19 app adoption. Thus, we ask:

RQ1: How do country-level factors (i.e., political system and the number of infected cases) influence the adoption of COVID-19 apps?

In addition to adoption, country-level factors can be potential predictors of evaluation. After downloading and using the apps, people would evaluate or rate them based on their experiences and feelings. In democratic societies, where there is higher freedom of speech and less self-censorship, people might be more willing to express their dissatisfaction and criticism. Therefore, apps in democratic countries are expected to receive a lower evaluation in terms of lower rating scores. But on the other hand, most democratic countries are developed with a much higher level of digital technology. Apps deployed in these countries could be more advanced, leading to a better user experience than those from less developed countries. Therefore, people are also likely to rate them higher. We thus ask:

RQ2: How do country-level factors (e.g., political system and the number of infected cases) influence the evaluation of COVID-19 apps?

Apart from country-level factors, another set of factors that might influence the adoption and evaluation of COVID-19 apps is app-level factors. In this study, we focus on the functions and developers of the apps. According to the uses and gratifications theory, media users use specific media to meet their desires and needs to achieve gratification [26]. In the scenario of COVID-19 apps, we can assume the user chooses a specific app because a particular set of functions that the app has would satisfy the needs of the user. Besides, whether the government or other institutions develop the app is also a critical factor for adoption. Research has found that a lack of trust in the government is one of the main barriers to adopting contact-tracing apps [11]. Therefore, whether or not the app is developed by a governmental institution would be very critical to the adoption rate. Based on the discussion above, we ask that:

RQ3: What is the influence of app-level factors (i.e., functions and developers) on the adoption of COVID-19 apps?

Since people would evaluate apps based on their experiences of using the apps, functions are also essential for evaluation. If people achieve gratification through using specific functions of an app, they might feel that the app is helpful for them and then give a high score. However, if an app’s function does not meet the need of users, it will be rated low. Some specific functions, such as contact tracing, are beneficial during the pandemic. Therefore, apps with the function of contact tracing might receive a higher score. For government-developed apps, given the varying levels of trust of different governments, the evaluation can vary accordingly. In sum, we ask:

RQ4: What is the influence of app-level factors (i.e., functions and developers) on the evaluation of COVID-19 apps?

Since the contact-tracing function of COVID-19 apps is currently on top of the research agenda, we also concern about the factors which are potentially crucial for the development of the contact-tracing function of an app. Therefore, we propose the following research question:

RQ5: What country-level and app-level factors will associate with the development of COVID-19 contact-tracing apps?

## Methods

### Data collection

**Fig. 1:**
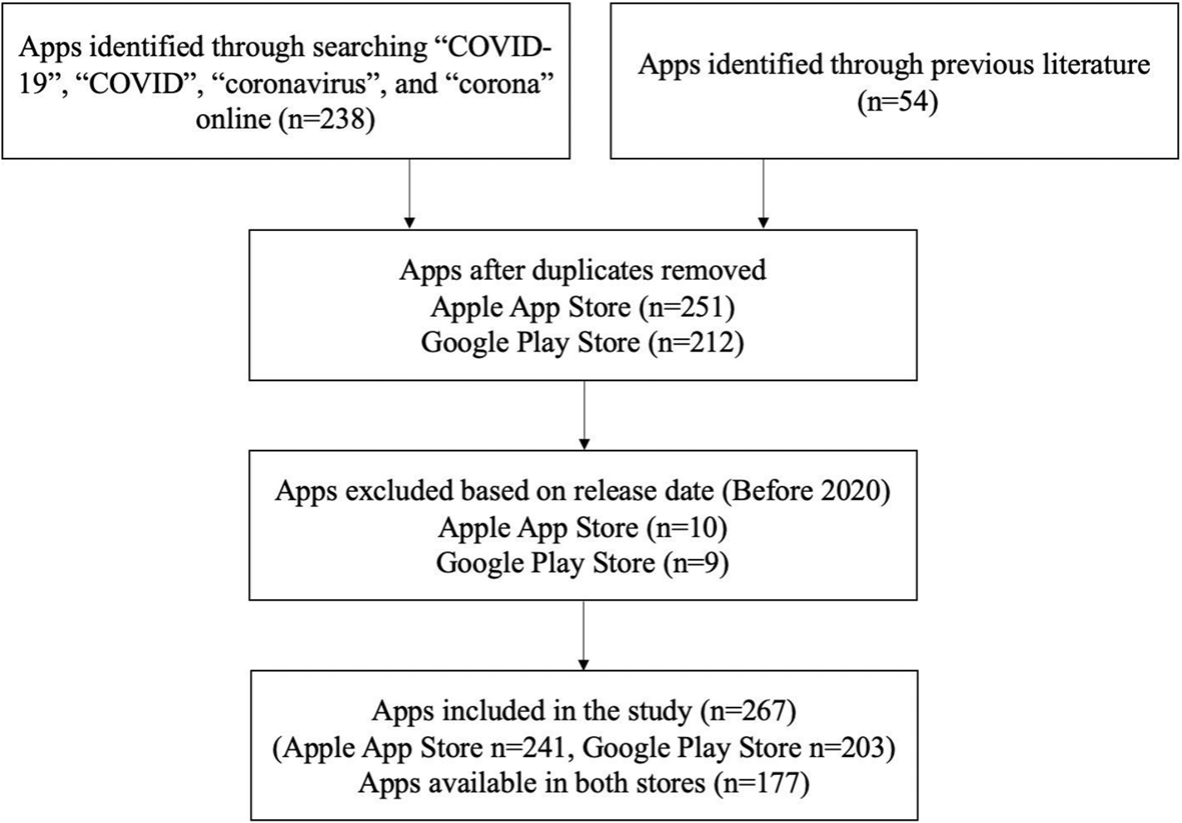
The selection of COVID-19 apps in the Apple App Store and the Google Play Store

The data of this study come from Apple App Store and Google Play. These two stores limit listing search results for “COVID-19” or “coronavirus,“ which is a barrier to identifying a full list of apps in the application stores. Therefore, we identified the name list of COVID-19 apps from two resources. For one, we identified app names from existing studies (5 studies) [19, 22, 27–29]. For the other, we used a website named fnd (https://fnd.io/) to search for relevant apps. This website provides search results on Apple App Store. The terms we used to search for apps are “COVID-19”, “COVID,“ “coronavirus,“ and “corona.“ Results from the two sources were then combined. In the next step, we deployed a Python program to crawl data from Google Play and Apple App Store based on the app name list. Data collection was conducted between January 20 and February 30, 2021. A total of 267 COVID-19 apps from 68 countries and global organizations (e.g., WHO) were collected. Of the 267 apps, 241 are from the Apple App Store, 203 are from the Google Play Store, and 177 are on both platforms. The selection process of the apps is shown in Fig. 1.

Data of the iOS-based Apple apps contains country, date of release, developer, description of functions, number of people rating the app, number of reviews, and star (review score of the app). Data of Android-based apps do not have the date of release but the number of installs. We identified the date of release of these apps from their counterparts in the Apple store, the institutions’ websites, news reports, or other web sources. All the apps were released between March 3, 2020, and December 20, 2020.

### Measures

Variables analyzed in this study were classified into outcome variables, country-level predictors, and app-level predictors.

#### Outcome variables

##### Number of installs

The number of installs refers to how many people downloaded and installed the app (M = 615366.13, SD = 1708263.90, Min = 5, Max = 10,000,000). Only Google Play records this number. Therefore, only apps in Google Play were included in the model for the number of installs.

##### Number of ratings

The number of ratings refers to how many people rated the app. Both platforms record this statistic. The variable was calculated by summing two numbers if the app exists on both platforms (M = 40690.57, SD = 274903.75, Min = 0, Max = 3,214,665). If the app only exists on one platform, we used the number on that platform for the variable.

##### Number of reviews

The number of reviews refers to how many people wrote reviews for the app. Both platforms record this statistic. The calculation of the variable followed the same process of calculation of the number of ratings (M = 611.92, SD = 2137.33, Min = 0, Max = 17,839).

##### Rating score

The rating score is the score users give to the apps. The range of the scores is between 1 and 5. Both platforms have a final score based on the rating of each user. The variable of rating was calculated by averaging the scores from both platforms. If the app has a rating score on only one platform, the score on the platform was used for the variable (M = 3.79, SD = 0.88).

#### Country-level factors

For each app, we identified which country or region it developed, and then we coded country-level variables for each app. Apps developed by a global institution such as WHO were excluded from the model. Country-level control variables include Internet users per capita, GDP, and population. Data on Internet users per capita were retrieved from the International Telecommunication Union (https://www.itu.int/en/ITU-D/Statistics/Pages/facts/default.aspx). The percentage of the online population was controlled in that a higher rate of Internet users indicates a higher level of digital technology, which may influence the adoption of COVID-19 apps. Data on population were collected from the Worldometer (https://www.worldometers.info/world-population). Data on GDP were collected from the World Bank Open Data website (https://data.worldbank.org/).

Country-level factors include the cumulated number of infections on the release date of the app in the country (referred to as the number of cases) and political system. The number of cases was collected from WHO (https://www.who.int). Political system was a dichotomous measure: democracy vs. non-democracy. It is coded based on the 2020 Democracy Index compiled by the Economist Intelligence Unit (EIU). Our data included 66 democratic and 12 non-democratic countries and regions.

#### App-level factors

Features and functions of an app were quantified as the app-level variables. The release date of the app was included as an app-level control variable. For ease of analysis, we transform the release date into four categories, respectively, before April 2020, April to June.2020, July to September 2020, and October to December 2020. We controlled release dates because apps released earlier could have better adoption and evaluation statistics. App-level independent variables include developers and functions.

##### Developer

A previous study [30] coded app developers into five categories: government, company, university, hospital, and NGO (non-governmental organization). We coded developer to be a dichotomous variable as government and non-government. In our data, there were 135 apps developed by the government and 132 apps by others.

##### Function

**Fig. 2:**
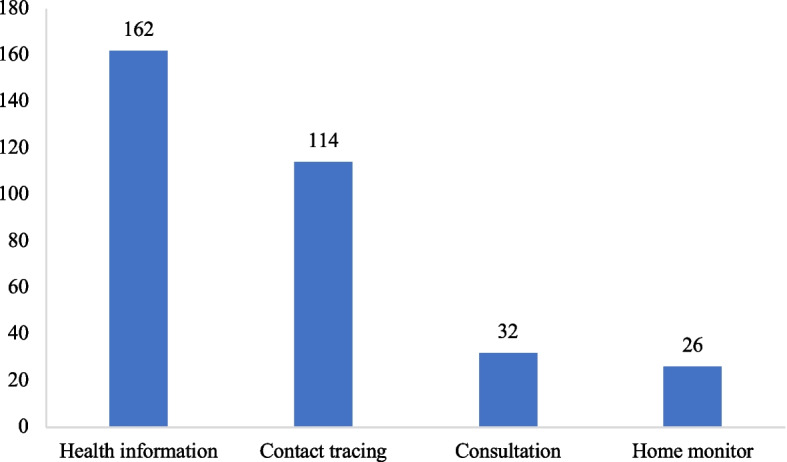
Category of functions of COVID-19 apps

Following previous studies, we identified four major functions based on the description of the apps: health information, contract tracing, home monitoring, and consultation. Health information means that the app provided COVID-19 information, such as news, policies, anti-epidemic knowledge, and self-assessment instruction. Contact tracing refers to the function that the app could notify users when they are possibly exposed to the COVID-19. Home monitoring is a function that monitors for a person in quarantine to ensure they stay in the place of quarantine. Consultation is a service provided by professional medicals who can communicate with users. The four functions were coded as four dummy variables. In our data, there were 162 apps with health information function, 114 apps with contact tracing function, 32 apps with consultation function, and 26 apps with home monitoring function. The descriptive analysis of app functions is shown in Fig. 2.

### Statistical analysis

In the current study, the unit of analysis is each app. First, negative binomial regression was used to estimate the impact of country-level and app-level predictors of the number of installs, number of ratings, and number of reviews of the apps. In our data, the distribution of the number of installs (M = 615366.13, SD = 1708263.90, Skewness = 4.37, Kurtosis = 19.91), number of ratings (M = 40690.57, SD = 274903.75, Skewness = 10.04, Kurtosis = 108.41), and number of reviews (M = 611.92, SD = 2137.33, Skewness = 5.67, Kurtosis = 36.38) were highly skewed toward 0. Therefore, negative binomial regression is chosen to analyze the over-dispersed count data where the conditional variance exceeds the conditional mean [31–33]. Second, OLS regression was used to estimate the impact of the same set of predictors on the rating score of the apps. Last, binomial logistic regression was conducted to estimate the impacts of country-level and app-level factors on the development of the contact-tracing function.

## Results

### Descriptive statistics

We first present some descriptive statistics of the data. For the release date, 49.06% of apps were released between April and June 2020, followed by 24.72% between July and September 2020. About 17.22% and 8.99% of apps were released before April 2020 and after October 2020, respectively. As of country of origin, the apps were developed by 60 governments and institutions globally. Among them, the U.S. has the highest number of apps (31.08%), followed by India (6.36%) and the U.K. (5.62%) (Table 1). For developers, government organizations were the principal developers of COVID-19 apps (50.56%).

**Table 1:** COVID-19 apps by country of origin (*N* = 267)

Country	*n* (%)
United States	83 (31.08)
India	17 (6.36)
United Kingdom	15 (5.62)
Canada	9 (3.37)
Australia, Mexico	7 (2.62)
Brazil	6 (2.24)
Italy, Malaysia, United Arab Emirates	5 (1.87)
France, Germany, Pakistan, Spain	4 (1.50)
Ireland, Netherlands, New Zealand, Russia, Saudi Arabia, South Korea, Switzerland, Vietnam	3 (1.12)
Czech Republic, Hong Kong, Indonesia, Morocco, Oman, Poland, Qatar	2 (0.75)
Argentina, Armenia, Bahrain, Belgium, Bolivia, Bulgaria, Colombia, Cyprus, Denmark, Ecuador, Estonia, Finland, Ghana, Hungary, Iceland, Israel, Jamaica, Japan, Jordan, Kuwait, Latvia, Liberia, Malta, Myanmar, North Macedonia, Norway, Portugal, Republic of Lithuania, Romania, Singapore, South Africa, Sri Lanka, Sweden, Thailand, The Dominican Republic, Turkey, Uganda, Ukraine, Uruguay	1 (0.37)

### Evaluation outcomes

For RQ1 to RQ4, negative binomial regression analysis was conducted to predict the number of installs (Model 1), the number of ratings (Model 2), and the number of reviews (Model 3). OLS regression was conducted to model the outcome variable of rating scores (Model 4). Results are shown in Table 2.

**Table 2:** Predicting number of installs, number of ratings, number of reviews, and rating scores of COVID-19 apps

	**Model 1 (Number of installs)**			**Model 2 (Number of ratings)**		
**Variables**	**IRR (S.E.)**	**95%CI**	***P*-value**	**IRR (S.E.)**	**95%CI**	***P*-value**
(Intercept)	423312.9 (2760158)	1.193, 1.50e+11	.047	0.010 (0.077)	0.000, 31031.920	.55
Release date	0.630 (0.186)	0.353, 1.125	.12	0.971 (0.551)	0.319, 2.951	.96
Population (log)	2.206 (0.792)	1.091, 4.460	.03	10.354 (6.273)	3.158, 33.945	<.001
GDP (log)	0.564 (0.250)	0.237, 1.342	.20	0.269 (0.159)	0.084, 0.859	.03
Internet user	1.020 (0.017)	0.986, 1.054	.25	1.115 (0.031)	1.056, 1.178	<.001
**Country-level factors**						
Number of cases (log)	0.917 (0.152)	0.663, 1.268	.60	0.716 (0.153)	0.470, 1.089	.12
Political system (Democratic=1)	0.772 (0.421)	0.264, 2.251	.64	0.925 (0.893)	0.140, 6.138	.94
**App-level factors**						
Developer (Government=1)	8.156 (3.654)	3.389, 19.626	<.001	26.036 (16.836)	7.331, 92.468	<.001
Function (Health information)	0.732 (0.261)	0.364, 1.472	.38	1.408 (0.749)	0.496, 3.995	.52
Function (Contact tracing)	4.533 (1.811)	2.072, 9.918	<.001	11.634 (7.154)	3.486, 38.827	<.001
Function (Home monitoring)	0.732 (0.389)	0.258, 2.075	.56	0.206 (0.155)	0.047, 0.896	.04
Function (Consultation)	4.885 (2.263)	1.970, 12.111	.001	17.194 (10.308)	5.309, 55.680	<.001
Log likelihood	-2177.551			-1470.949		
LR chi2	72.42			74.54		
Pseudo R2	1.64			2.47		
R-squared						
N	169			169		
	**Model 3 (Number of reviews)**			**Model 4 (Rating score)**		
**Variables**	**IRR (S.E.)**	**95%CI**	***P*-value**	**Coefficient (S.E.)**	**95%CI**	***P*-value**
(Intercept)	2.919 (19.028)	0.000, 1031620	.87	6.255 (1.379)	3.537, 8.974	<.001
Release date	0.277 (0.101)	0.136, 0.565	<.001	-0.049 (0.107)	-0.261, 0.163	.65
Population (log)	0.258 (0.141)	0.088, 0.754	.01	0.150 (0.121)	-0.088, 0.388	.22
GDP (log)	3.137 (1.562)	1.182, 8.324	.02	-0.215 (0.109)	-0.430, 0.001	.05
Internet user	0.951 (0.026)	0.902, 1.002	.06	0.007 (0.006)	-0.005, 0.019	.24
**Country-level factors**						
Number of cases (log)	1.081 (0.193)	0.763, 1.533	.66	0.079 (0.040)	0.000, 0.158	.049
Political system (Democratic=1)	0.548 (0.427)	0.119, 2.526	.44	-0.220 (0.222)	-0.657, 0.217	.32
**App-level factors**						
Developer (Government=1)	12.188 (7.000)	3.954, 37.568	<.001	-0.369 (0.145)	-0.653, -0.083	.01
Function (Health information)	1.314 (0.556)	0.573, 3.013	.52	0.032 (0.126)	-0.216, 0.281	.80
Function (Contact tracing)	5.688 (2.959)	2.052, 15.770	.001	-0.072 (0.142)	-0.352, 0.208	.61
Function (Home monitoring)	3.871 (2.588)	1.044, 14.349	.04	-0.550 (0.214)	-0.971, -0.129	.01
Function (Consultation)	16.718 (9.698)	5.363, 52.113	<.001	0.292 (0.182)	-0.066, 0.650	.11
Log likelihood	-938.135					
LR chi2	58.00					
Pseudo R2	3.00					
R-squared				13.86		
N	170			216		

Model 1 presents an analysis predicting the number of installs. The number of installs is only available in Google Play. It is an important indicator of app adoption. After controlling for the release date, population, GDP, and Internet user per capita, none of the country-level factors significantly predicted the number of installs. However, the IRR values showed that apps developed by governments would be more likely to be installed by a factor of 8.156 than apps developed by other institutions. Apps with functions of contact tracing and consultation were more likely to be installed by factors of 4.533 and 4.885, respectively, compared to apps without these functions. The result indicates that people tend to download and install apps developed by the government or apps with contact tracing and consultation functions.

In Model 2, the pattern of the result was similar to those of Model 1. Although none of the country-level factors predicted the number of ratings, app-level factors, including government developers, functions of contact tracing, home monitoring, and consultation, were all significantly associated with the number of ratings. For app-level factors, apps developed by governments were more likely to receive higher numbers of ratings than apps developed by other institutions. The incident rate ratio (IRR) value shows that compared to non-governmental institutions, apps developed by governments would be more likely to receive higher ratings by a factor of 26.036. Among various functions, functions of contact tracing, home monitoring, and consultation were all significantly associated with the number of ratings. The incident rate ratio (IRR) values showed that apps with functions of contact tracing and consultation were more likely to receive more ratings by factors of 11.634 and 17.194, respectively. In comparison, apps with the function of home monitoring were likely to receive fewer ratings by a factor of 0.206, compared to apps without these functions. It means that people are more likely to rate apps developed by governments or with functions of contact tracing and consultation. Meanwhile, they are less likely to rate apps with the function of home monitoring.

Model 3 is about factors predicting the number of reviews. It showed a similar pattern as Model 2 suggested. After controlling for the same control variables, none of the country-level factors significantly predicted the number of reviews. For app-level factors, the incident rate ratio (IRR) value showed that compared to non-governmental institutions, apps developed by governments were more likely to receive more reviews by a factor of 12.188. Apps with functions of contact tracing and consultation were more likely to receive more reviews by factors of 5.688 and 16.718, respectively. Apps with home monitoring functions were also more likely to receive a higher number of reviews, by a factor of 3.871, compared to apps without this function. In other words, people tend to give reviews to apps developed by government organizations or apps with functions of contact tracing, home monitoring, and consultation.

Model 4 predicts rating scores. Since rating score is a continuous measure, OLS regression was used to analyze the data. The data showed that number of cases was positively associated with rating scores (B = 0.079, CI = 0.000, 0.158; *P* = .049). The higher number of infected patients there were in a specific country, the apps would be rated as more helpful. For app-level factors, apps developed by governmental organizations received lower rating scores (B=-0.369, CI=-0.653, -0.083; *P* = .01). Besides, home monitoring was significantly related to the rating score (B=-0.550, CI=-0.971, -0.129; *P* = .01). Apps with the function of home monitoring received lower scores than apps without this function. In sum, the results showed that users prefer giving low scores to apps with home monitoring functions or apps developed by government organizations.

To answer RQ5, we performed binominal logistic regression to identify the country-level and app-level predictors of the contact-tracing function. Results were shown in Table 3. Political system and developer were significant predictors. In democratic countries, COVID-19 apps were more likely to have contact tracing (OR = 3.650, CI = 1.238, 10.758; *P* = .02). In addition, government-developed apps were more likely to include this function (OR = 7.987, CI = 4.106, 15.534; *P* < .001).

**Table 3:** Predicting contact tracing

	**Contact tracing**		
**Variables**	**Odds Ratio (S.E.)**	**95% CI**	***P*-value**
(Intercept)	0.001 (0.002)		.03
Release date	1.524 (0.404)	0.906, 2.563	.11
Population (log)	0.725 (0.220)	0.400, 1.316	.29
GDP (log)	1.477 (0.413)	0.854, 2.553	.16
Internet user	1.011 (0.015)	0.891, 1.041	.48
**Country-level factors**			
Number of cases (log)	0.831 (0.082)	0.685, 1.008	.06
Political system (Democratic=1)	3.650 (2.013)	1.238, 10.758	.02
**App-level factors**			
Developer (Government=1)	7.987 (2.711)	4.106, 15.534	<.001
Log likelihood	-141.822		
Pseudo R2	17.45		
N	251		

For ease of understanding, we summarized all our findings and displayed them in Table 4.

**Table 4:** Summary of analytical findings

DV IV	**Number of installs**	**Number of ratings**	**Number of reviews**	**Rating score**	**Contact tracing**
*Country-level factors*					
Number of cases	n.s	n.s	n.s	+	n.s
Political system	n.s	n.s	n.s	n.s	+
*App-level factors*					
Developer	+	+	+	−	+
Health information	n.s	n.s	n.s	n.s	n.a
Contact tracing	+	+	+	n.s	n.a
Home monitoring	n.s	+	+	−	n.a
Consultation	+	+	+	n.s	n.a

"+" denotes a significant positive relationship; "-" denotes a significant negative relationship

## Discussion

This study analyzed existing COVID-19 apps to explore factors influencing the adoption and evaluation of these apps. We used number of installs, number of ratings, and number of reviews as indicators of adoption and rating score as the indicator of evaluation. Although we did not directly measure adoption and evaluation at the individual level, the data we used served as macro-level indicators regarding people’s behaviors towards the COVID-19 apps, which helps us to understand the use of these apps during the pandemic.

### Number of the infected case and rating score

Regarding country-level factors, the number of infected cases in a country was associated with the rating score of the apps. Specifically, the higher the number of infected cases in a country, the higher score COVID-19 apps would receive. By reading some app reviews from countries with the highest numbers of infected cases, we found that citizens use these apps for different purposes. For example, in India, which has the second-highest number of infections in the world, several apps receive high scores. One of the apps, named AarogyaSetu was developed for contact tracing, which was highly rated by the users. Users found the app an “effective way of containing COVID-19” and “keeping them safe.“ Another highly rated app named Covid SafePass was used as a Covid-19 test passport. People can upload their test results and provide the test results to co-workers or friends when they recover. Another app “Corona Checker” was used for self-diagnosis. Many users thought it valuable and helpful because it can provide some recommendations before going to the hospital. In short, some of the COVID-19 apps were found to be valuable by users in countries with high infection rates, such as India.

### Government-developed apps and adoption, rating score

Regarding app-level factors, whether an app was developed by government organizations seems to be a decisive factor for its adoption and evaluation. Apps developed by governments were more likely to be installed, to receive more ratings and reviews, but with lower ratings. During international crises, trust in government is expected to increase [34, 35]. During the COVID-19 pandemic, trust in the government was found to increase dramatically in some countries [36, 37]. Trust in the government strongly predicted COVID-19 phone application use, mainly by convincing people that COVID apps are beneficial [36]. In our study, evidence shows that people were more likely to download the apps developed by governmental organizations. This finding provides indirect evidence that trust in the government increased during the pandemic. Another reason that apps developed by governments were adopted more frequently is that some apps were mandatory by government policies. And some are used as proof of pass with an individual’s COVID-19 test result along with vaccination records in the apps. Individuals must use the apps to enter public spaces such as restaurants, working places, schools, or hospitals. Most of these apps were developed by the government. For example, the app TraceTogether by the Singapore government was a necessary tool for people living in Singapore. It was downloaded in Google Play one million times by the date we collected the data. The app allows individuals to present COVID health status based on their vaccination and test statuses before entering public spaces. Apps of this kind are necessary for people’s daily life.

While apps developed by the government were adopted more, they received lower rating scores. We have limited information to make sense of this result with the empirical evidence collected in the current study. However, we speculate that users could have some concerns with these government developed apps, such as privacy protection and maintenance quality.

### Contact tracing, consultation, and adoption

App functions are critical for app adoption. We found that only functions of contact tracing and consultation significantly predict adoption. Apps with these two functions would be downloaded more times, as well as receive more ratings and reviews. Contact tracing is a very important function developed for the COVID-19 pandemic. The app requires users to submit personal information and to turn on Bluetooth or GPS. Using these technologies, governments and other institutions can identify, evaluate, notify, and manage users who are exposed to COVID-19 [38]. In addition, health authorities can trace the whereabouts of close contacts through these apps. The contact tracing function supplements the functions of news media, which can broadcast the status of the epidemic and inform people about how to prevent the virus. Although the privacy issue related to contract tracing is still under debate, more and more people, no matter in democratic or non-democratic countries, are willing to use this function and, at the same time, give up some of their privacies [18]. The function of consultation helps people to communicate with health professionals. It is useful during the epidemic to meet the needs of ordinary people. They can communicate with health professionals without visiting the hospital, reducing the risk of infection.

### Home monitoring and adoption, evaluation

We find that the function of home monitoring was associated with both the adoption and evaluation of apps. Apps with the home monitoring function would receive fewer ratings, more reviews, and a lower rating score. Home monitoring is a function developed mainly by the government to monitor a person in quarantine to ensure they stay in the quarantine place. Therefore, these apps were mostly developed for the only purpose and mostly downloaded mandatorily. For example, the app StayHomeSafe was deployed by the Hong Kong Government to monitor a person during home quarantine. It was required to be installed when entering Hong Kong. The app Home Quarantine – Poland was required for entering Poland, and the app Quarantine Report – Self-Check was required for entering South Korea. Our analysis results showed that people usually gave these apps a lower rating score.

### Limitations

This study has a few limitations. First, although we collected data on adoption and evaluation, we could not distinguish whether the users use these apps voluntarily or by compulsory order. Second, due to the constraints of searching directly in application stores, we obtained the list of the apps mainly from searching an Apple Store archive. Therefore, the list is by no means exhaustive. Third, for ease of analysis, we only coded four major COVID app functions. Other functions, such as mental health monitoring and proof of pass were not included in our study. Last but not least, for evaluation, we only used rating scores as the quantitative indicator. But the detailed comments about the apps are more informative. Further studies could consider analyzing comment text.

## Conclusion

The COVID-19 pandemic is still impacting the world. Many mobile apps related to COVID-19 were developed for different purposes during the pandemic. This study offers a systematic investigation into factors associated with the adoption and evaluation of COVID-19 apps. Evidence shows that whether the app was developed by the government, as well as the functions of contact tracing and consultation, strongly predicts app adoption. The results of the study might provide insights into the understanding of mobile app use during a pandemic.

## Data Availability

The datasets used and analyzed during the current study are available from the corresponding author on reasonable request.

## Abbreviations

CI: confidence interval
COVID-19: coronavirus disease
EIU: Economist Intelligence Unit
IRR: incident rate ratio
OLS: ordinary least square
OR: odds ratio
S.E.: standard error
WHO: World Health Organization
